# Cardioprotective Peptides from Milk Processing and Dairy Products: From Bioactivity to Final Products including Commercialization and Legislation

**DOI:** 10.3390/foods11091270

**Published:** 2022-04-27

**Authors:** Armin Mirzapour-Kouhdasht, Marco Garcia-Vaquero

**Affiliations:** School of Agriculture and Food Science, University College Dublin, Belfield, Dublin 4, D04 V1W8 Dublin, Ireland; armin.mirzapourkouhdasht@ucd.ie

**Keywords:** bioactive peptides, cardioprotective peptides, dairy processing, dairy products

## Abstract

Recent research has revealed the potential of peptides derived from dairy products preventing cardiovascular disorders, one of the main causes of death worldwide. This review provides an overview of the main cardioprotective effects (assayed in vitro, in vivo, and ex vivo) of bioactive peptides derived from different dairy processing methods (fermentation and enzymatic hydrolysis) and dairy products (yogurt, cheese, and kefir), as well as the beneficial or detrimental effects of the process of gastrointestinal digestion following oral consumption on the biological activities of dairy-derived peptides. The main literature available on the structure–function relationship of dairy bioactive peptides, such as molecular docking and quantitative structure–activity relationships, and their allergenicity and toxicity will also be covered together with the main legislative frameworks governing the commercialization of these compounds. The current products and companies currently commercializing their products as a source of bioactive peptides will also be summarized, emphasizing the main challenges and opportunities for the industrial exploitation of dairy bioactive peptides in the market of functional food and nutraceuticals.

## 1. Introduction

Bioactive peptides can be described as short chains of amino acids (2–20 residues) with strong health-promoting properties [1,2,3]. Generally, processed milk (hydrolysis or fermentation) and dairy products (cheese, yogurt, and kefir) can be considered as excellent sources of bioactive peptides. These peptides are inactive within the intact structure of dairy proteins and they gain strong biological activities once they are released by different hydrolysis methods [4], such as the in vitro fermentation of milk with a proteolytic starter during dairy processing or also in vivo after ingesting proteins during the process of gastrointestinal (GI) digestion [5,6]. Thus, the production of bioactive peptides can be considered as an expanded business for the dairy industry as co-products such as whey protein, with an annual production of approximately 180–190 million tons [7], can be excellent sources for the generation of bioactive peptides.

**Figure 1:**
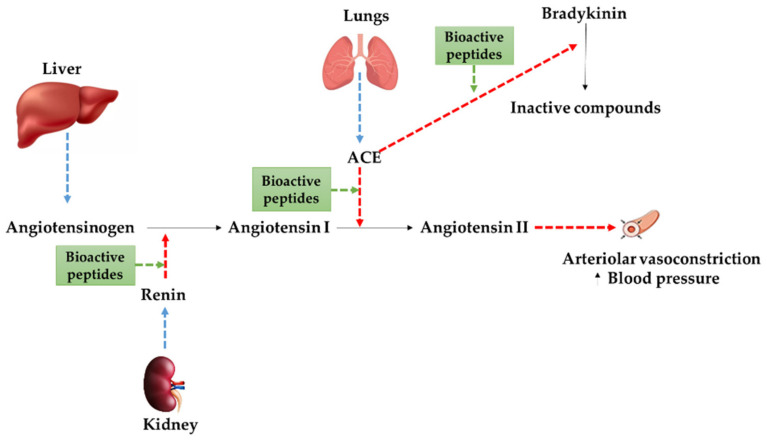
Potential mechanisms for the cardioprotective effects of bioactive peptides derived from dairy.

Bioactive peptides have shown very strong health-promoting properties, being capable of lowering the risks of many diseases, such as cardiovascular disorders (CVD) [1,2,3]. CVD is a global term to encompass multiple disorders including heart failure, atherosclerosis, cerebrovascular disease, peripheral vascular disease, and other cardiac anomalies [8], and it is considered as the most common cause of death in the general population globally every year [9]. Lowering high blood pressure or hypertension is a prominent target to prevent CVD. There are two different mechanisms for lowering blood pressure: following the renin–angiotensin system (RAS), the enzyme renin catalyzes the conversion of angiotensinogen to angiotensin I. Following a cascade reaction, the angiotensin-converting enzyme (ACE) subsequently converts angiotensin I to angiotensin II which is a vasoconstrictor agent that will ultimately generate an increased high blood pressure. In addition, ACE has a role in the kinin–nitric oxide system (KNOS) converting the vasodilator agent bradykinin into inactive components [10]. The inhibition of both renin and ACE by bioactive compounds, including bioactive peptides, could be used as a promising targeted strategy to control CVD. Thus, dairy products and derived bioactive peptides can be an excellent candidate in the prevention of these disorders as a source of bioactive peptides [11,12,13] with multiple targets to exert these cardioprotective activities as summarized in Figure 1.

To date, several reviews on milk products and bioactive peptides have focused specifically on methods to produce and analyse bioactive peptides [14,15,16] or on the multiple health benefits related to the consumption of these peptides [15,17] without deeper explanation on the mechanism of action of the compounds or the effect of gastrointestinal digestion on their biological properties when these compounds are administered as food. The current work aims to review the state of the art and main bioactive peptides with anti-hypertensive activities in vitro, in vivo, and ex vivo discovered from milk processing (enzymatic and microbial hydrolysis) and dairy products (milk, yogurt, cheese, and kefir) as well as the effects of GI digestion on their structure. Moreover, the structure–function relationship of these bioactive peptides, as well as studies evaluating their allergenicity and toxicity, will also be reported. The products currently in the market as a source of bioactive peptides and the legislative frameworks available worldwide that regulate the commercialization of these compounds will also be summarised aiming to establish a clear scenario for the future use and exploitation of these peptides by the dairy industry.

## 2. Bioactive Peptides Derived from Milk

Bioactive peptides from milk are normally sequences of 2–50 amino acids [18] generated by in vivo and/or in vitro digestion processes, displaying cardioprotective effects upon their release from casein [4]. These peptides may contribute to lower the risk of CVD through several pathways as previously described in Figure 1. These routes for CVD prevention include: (i) inhibiting the renin secreted from the kidney; (ii) inhibiting the conversion of angiotensin I into angiotensin II; and (iii) inhibiting the conversion of bradykinin to inactive components (non-vasodilator agents) [10,19].

Milk production worldwide has continued to grow over the last decade: 497 million metric tons of cow milk were produced in 2015, rising to levels of up to 544 million metric tons by 2021 [20]. The main proteinaceous constituents of milk that could be used for the generation of cardioprotective peptides include caseins (αS1-casein, αS2-casein, β-casein, and k-casein), α-lactalbumin (α-LA), β-lactoglobulin (β-LG), immunoglobulins, lactoferrin, protease–peptide fractions, serum albumin, and transferrin [21]. These proteins could be used for either in vivo or in vitro digestion to generate bioactive peptides and then purify them by different separation techniques to assess their cardioprotective effects.

Different factors affect the cardioprotective effects of bioactive peptides from milk, including the protein source, peptide sequence, and proteolytic activity. The hydrolysis process of milk by proteolytic enzymes is currently the preferred approach to obtain cardioprotective bioactive peptides from dairy proteins. The advantages of this method include high reproducibility, target specificity and controllable conditions during the hydrolysis [22], resulting in a cost-efficient process really suitable for the generation of these compounds at industrial level. There are several studies researching the cardioprotective effects of bioactive peptides derived from dairy proteins following a hydrolysis process [23,24,25,26,27]. The hydrolysis conditions can affect the bioactive peptides’ production efficiency as well as their cardioprotective activity. Four major factors of the enzymatic hydrolysis process have been introduced to control the cardioprotective effects of generated bioactive peptides derived from dairy products including hydrolysis temperature, time, pH, and enzyme to substrate ratio. In a research performed by Guo et al. [28], the optimum hydrolysis conditions for ACE inhibitory of whey protein concentrate hydrolysates (obtained by crude proteinases from *Lactobacillus helveticus* LB13) were determined by response surface methodology (RSM). The results of this study indicated that the optimum conditions for achieving ACE inhibitory activity of 92.2% were an enzyme to substrate ratio of 0.60, 8 h, pH of 9.18, and 38.9 °C of temperature. The degree of hydrolysis is a factor that is positively related to the cardioprotective activity of the bioactive peptides [29]. This degree of hydrolysis is normally increased by increasing the processing time, temperature, and pH until certain levels specific for each protein in which no further degree of hydrolysis is appreciated due to the denaturation of the hydrolytic enzymes under unfavorable conditions [30]. The enzyme to substrate ratio is not linearly related to the degree of hydrolysis and cardioprotective effects of the peptides [31]. This effect could be due to an enzymatic steric effect that does not allow contact between the protein and the catalytic sites in the enzymes, and the reduction of substrate diffusion and saturation reaction rates [30]. In a study performed by Mazorra-Manzano et al. [32], whey protein was hydrolyzed to generate ACE inhibitory peptides using plant proteases. Whey protein hydrolysates (especially those from β-lactoglobulin) revealed the highest ACE inhibitory of 75–90%. However, the authors did not determine the amino acid sequences of the bioactive peptides from these hydrolysates responsible for that effect.

The peptide sequences discovered from different milk proteins using proteolytic enzymes, as well as the cardioprotective activities reported in the scientific literature, are summarized in Table 1. Lin et al. [33] used qula casein from yak milk and hydrolyzed it using different enzymes (alcalase, α-chymotrypsin, thermolysin, proteinase K, trypsin, and papain). The authors identified 3 bioactive peptides with ACE inhibitory activity in vitro, PFPGPIPN, KYIPIQ, and LPLPLL, with IC_50_ of 12.79, 7.28, and 10.46 μM, respectively [33]. Lin et al. [34], indicated that qula casein hydrolysed by two approaches (combination of thermolysin + alcalase and thermolysin + proteinase K) could be a source of ACE inhibitory peptides. The identified bioactive peptides (KFPQY, MPFPKYP, MFPPQ, and QWQVL) were chemically synthesized, among which the highest ACE inhibitory activity, with IC_50_ of 12.37 μM, of the peptide KFPQY.

The generation of bioactive peptides from the fermentation process could be a prominent approach to obtain peptides with potential cardioprotective effects. These bioactive peptides could be released from milk fermentation using different generally recognized as safe (GRAS) microorganisms, such as lactic acid bacteria (LAB). Milk proteins are a good nitrogen source for proteolytic enzymes from starters, generally LAB, generating cardioprotective peptides during dairy processing [44,45,46]. A comprehensive list of cardioprotective bioactive peptides derived from the milk fermentation process using different microbial cultures is listed in Table 2.

Li et al. [47] investigated the influences of co-cultures of *Lactobacillus plantarum* and *Bifidobacterium animalis* ssp. *lactis* combined with *Streptococcus thermophiles* for the production of bioactive peptides from fermented milk and the ACE inhibitory activity of these peptides in vitro. The authors determined that *Streptococcus thermophiles* combined with *Lactobacillus plantarum* and/or *Bifidobacterium animalis* increased production of bioactive peptides and ACE inhibitory activity compared to *Streptococcus thermophiles* alone.

**Table 2:** Cardioprotective peptides derived from milk using fermentation proteolytic procedures.

Original Proteins	Microbial Cultures	Peptide Sequences	References
β-casein and k-casein	*Lactobacillus helveticus* and *Saccharomyces cerevisiae*	VPP and IPP	[48]
β-casein and k-casein	*Lactobacillus helveticus* LBK16H	VPP and IPP	[49]
β-casein and α_S1_-casein	*Lactobacillus GG* and enzymes + pepsin and trypsin	YPFPAVPYPQRTTMPLW	[50]
Whey proteins	*Lactobacillus helveticus* CPN 4	YP	[51]
β-casein	*Lactobacillus rhamnosus* + digestion with pepsin and Corolase PP	DKIHPFYQEPVLVKEAMAPK	[52]
β-casein	*Lactobacillus delbrueckii* ssp. *bulgaricus*	SKVYPFPGPI	[53]
β-casein	*Streptococcus salivarius* ssp. *thermophiles* + *Lactococcus lactis* biovar. *diacetylactis*	SKVYP	[53]
β-lactoglobulin	*Kluyveromyces marxianus* var. *marxianus*	YLLF	[54]
β-casein	*Enterococcus faecalis* CECT 5727	LHLPLP and LVYPFPGPIPNSLPQNIPP	[55]
Whole milk	*Enterococcus faecalis* TH563 and *Lactobacillus delbrueckii* subsp. *bulgaricus* LA2	Not identified	[56]
Whole milk	*Bifidobacterium bifidum* MF 20/5 and *Lactobacillus casei* YIT 9029	Not identified	[57]
β-casein	*Enterococcus faecalis* BCS27	VVVPPF and ENLLRF	[58]
β-casein	*Bifidobacterium bifidum* MF 20/5	LVYPFP	[59]
κ-casein and αs2-casein	*Kluyveromyces marxianus* Z17	VLSRYP and LSFF	[60]
Whole goat milk	Wild *Lactobacillus plantarum* 69	Not identified	[61]
β-casein derived from camel milk	*Leuconostoc lactis* PTCC1899	MVPYPQR	[62]

Camel milk has been consumed traditionally for its biological benefits in traditional medicine, however, the knowledge of the mechanisms of action for these health benefits have remained under-explored to date. During the fermentation of camel milk, different researchers reported the release of bioactive peptides. Recent studies have shown the ACE-inhibitory activity of peptides released during the fermentation of camel milk with *Lactobacillus rhamnosus* MTCC 5945 as indicated by Solanki and Hati [63]. These authors identified nine peptides, MQTDIMIFTIGPA, VRTPVTVQTKVDNIKKY, EQAGRQRQGG, VRTPVTVQTKVDNIKKY, AXEAIFGAVVXIDL, VIAGGCAAIIG, GLVASIPR, QCDIMIFTIGPA, and VNPNTPIR, all of which show high ACE inhibitory activities [63].

It is worth exploring the differences between the cardioprotective effects of bioactive peptides derived from the fermentation of milk obtained from various animal sources including bovine, camel, goat, and so on. However, there is currently no information or scientific reports on this aspect. Further research will be needed to achieve a comprehensive knowledge of this topic. In silico procedures could aid researchers to achieve preliminary findings predicting the peptides released from known and sequenced milk proteins. In a research, goat milk, a perfect food source similar to human milk, was analyzed in silico by performing hydrolysis with pepsin and chymotrypsin A to obtain some bioactive peptides with ACE inhibitory activity. Several peptides of variable length (di-to undeca-peptides) were identified from α-S1, α-S2, and β casein fractions [64].

## 3. Bioactive Peptides from Yogurt

**Figure 2:**
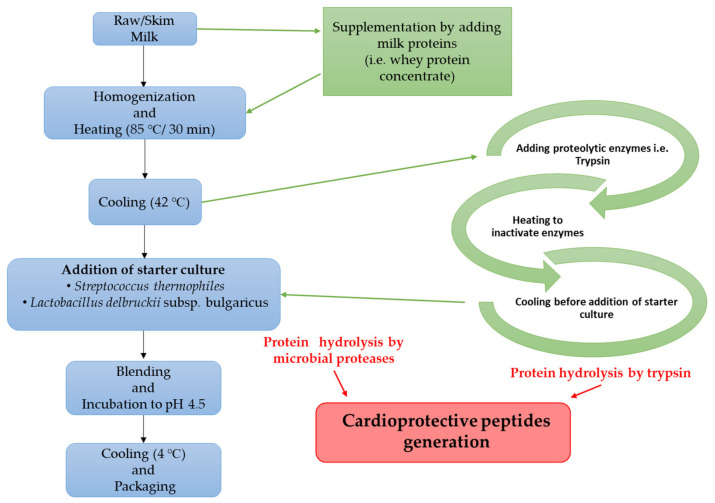
Generation of cardioprotective peptides during yogurt production process.

Milk fermentation during yogurt production by LAB has been researched as a cost-efficient and practical procedure to obtain bioactive peptides with cardioprotective activities [65,66] as summarized in Figure 2. As depicted in this figure, the incorporation of raw milk with additional proteins (i.e., whey protein concentrate) and proteolytic enzymes before the addition of a starter culture results in enhanced generation of cardioprotective peptides.

Regular consumption of yogurt has been linked to decreased risk of high blood pressure (17%) and CVD (21%) [67], which is directly related with the contents of bioactive peptides generated either by enzymatic hydrolysis or during the fermentation process as well as during the GI digestion of the products [68,69,70].

There are many ACE-inhibitory bioactive peptides discovered from yogurt as well as other peptides that can exert an antihypertensive activity through other mechanisms including inhibition of endothelin-1 release [71], enhancement of vasodilation activity of bradykinin [72], improvement of nitric oxide generation from endothelium [49], and binding to opiate receptors to stimulate the vasodilatory activity [66,73].

Among the large volume of research performed to discover cardioprotective peptides from fermented dairy products [44,45,46,47,62,63,74], only a few studies have reported these peptides from yogurt [66,75,76,77]. Milk fermentation using LAB could be considered as a prominent procedure to generate cardioprotective bioactive peptides from proteins, especially caseins [74]. It is reported that VPP and IPP are the two main bioactive peptides generated in yogurt, with a similar mechanism of ACE inhibition to that of synthetic drugs, such as captopril and lisinopril [77]. Increasing the protein content and using proteolytic bacteria strains in yogurt led to the release of the peptides αS1-casein f(24–32) and β- casein f(193–209) with ACE-inhibitory activity [75]. Papadimitriou et al. [76] prepared two types of traditional Greek sheep yogurt and analyze the physicochemical and cardioprotective properties of the products. One yogurt was made using a normal culture of *Lactobacillus delbrueckii* subsp. *bulgaricus* ϒ10.13 and *Streptococcus thermophilus* ϒ10.7, while the other yogurt type included *Lactobacillus paracasei* subsp. *paracasei* DC412. The authors identified the peptide YPVEPFTE with high ACE-inhibitory activity (IC_50_ 0.37 mg/mL), derived from β-casein 114–121. This peptide was also identified from γ-casein [78]. Further research into this peptide revealed that its cardioprotective mechanism of action was related to the inhibition of ACE and to blocking bradykinin-degrading enzymes [72]. Additional research is needed to clarify the cardioprotective effects and mechanisms of action of bioactive peptides in yogurt produced by various starter cultures, as well as the differences in microbial proteolytic systems that could lead to countless bioactive peptides enriching the final products.

## 4. Bioactive Peptides from Cheese

Bioactive peptides are prominent components in different types of cheese. The application of advanced peptidomic approaches using techniques, such as mass spectrometry in combination with bioinformatics-based procedures, demonstrated that thousands of bioactive peptides could be generated during the cheese making process [79,80]. Bioactive peptides contribute not only to cheese flavor and texture, but also reveal various health-promoting activities, such as cardio-protection, immunomodulation, and antimicrobial and antioxidant properties [81,82,83,84].

Amongst all the cardioprotective peptides described in the literature, the peptides IPP and VPP have been identified in 36 kinds of cheese. VPP concentration ranged from 0 to 224 mg/kg cheese, while IPP contents varied from 0 to 95.4 mg/kg cheese, with the highest concentrations of both (224.1 and 95.4 mg/kg for VPP and IPP, respectively) identified in the hard cheese “Hobelkäse from the Bernese Oberland” that also had the highest ACE-inhibitory activity (IC_50_ 2.6 mg/mL) [85]. The differences appreciated in the contents of these bioactive peptides relate to processing conditions including raw milk pre-treatments, the microbial cultures applied as well as the scalding and ripening conditions. Hence, it is highly recommended to generate a reproducible process to produce cheese containing high and constant concentrations of these bioactive peptides that could be enter the market of nutraceuticals [86].

Several studies describing and identifying bioactive peptides in multiple varieties of cheese are summarized in Table 3. From all these data reported in the literature, it can be concluded that there are several factors affecting the production of cardioprotective peptides during the cheese-making process including the type of cheese, microbial cultures used, and the original protein sources. Moreover, the ripening process has also strongly influenced the generation of peptides and the health-enhancement benefits of the final products [12]. Ripening is a prominent step in cheese making that relates specifically to the enzymatic coagulation process and enhances the flavor and texture properties of the final product. Several biochemical reactions—including proteolysis, lipolysis, glycolysis, and free fatty acids catabolism [87]—can affect the quality of the cheese, with proteolysis being the most relevant for the purpose of generation of bioactive peptides. The main agents responsible for the proteolytic process during ripening include enzymes from residual coagulant, plasmin from milk, microbial enzymes secreted from primary and secondary cultures or bacterial contaminants, as well as proteases appended to speed up the ripening process [87,88]. The generation of bioactive peptides is likely to happen mainly at an early stage of cheese ripening as the residual coagulant and plasmin hydrolyze caseins to form large and medium-size peptides. Subsequently, other proteolytic agents come apart to generate small bioactive peptides and amino acid residues with cardioprotective effects [18,88].

Atanasova et al. [95] researched the presence of cardioprotective peptides and free amino acids in Bulgarian goat, sheep, and cow white brined cheeses, generated using the same microbial culture. The main bioactive peptides with ACE inhibitory activities were identified from αS1 (RPKHPIKHQ.G, RPKHPIKHOGLPQEVLN.E, K.HPIKHQ.G, V.APFPEVF.G, L.KKYKVPQL.E, and Y.KVPQL.E) and β-CN (Q.DKIHPF.A). The authors also mentioned that no cardioprotective peptide were identified from αS2. In another research, more than 100 bioactive peptides with potential cardioprotective effects were identified from Gouda cheese using in silico and in vitro approaches [96]. As the authors illustrated, the identified bioactive peptides were encrypted in different casein sequences including αS1, αS2, β, and κ. Altogether, bioactive peptides derived from different types of cheese obtained by various processing procedures can be used as cardioprotective agents, there are limited studies evaluating the presence of bioactive peptides in different types of cheese using multiple bacterial cultures specific for those cheese varieties. Moreover, there is still research needed to evaluate the consistent presence of these compounds as well as their availability and effectiveness in vivo.

## 5. Bioactive Peptides from Kefir

Kefir is a beverage with a long history of production and consumption, with sour and slightly alcoholic flavor manufactured from milk fermentation originally in the Caucasian Mountains. This product, which is a result of milk fermentation by a mixed microflora in kefir grains, is different in composition and sensory properties to fermented milk [97,98,99]. Evidence has shown that the cardioprotective effects of kefir are related to the presence of bioactive peptides generated during fermentation process of this product. In a study performed by Şanli et al. [100], the effects of adjunct microbial cultures on ACE inhibitory and peptide profile of kefir were examined. This study revealed that prolonged storage of kefir fermented with *Lactobacillus helveticus* resulted in products with potent ACE inhibitory activity. These findings were confirmed by multiple authors fermenting kefir with *Lactobacillus helveticus* [101,102]. Shu et al. [103] indicated that goat milk kefir has cardioprotective effects due to the proteolytic activity of microflora of the kefir grains on the casein, generating bioactive peptides with antihypertensive properties. Moreover, the authors also confirmed that the generation of these peptides continued during the storage of the product, leading to changes in the structure of bioactive peptides and, thus, the ACE-inhibition rate of kefir. Amorim et al. [104] researched the cardioprotective mechanism of action of kefir by performing a tryptic digestion of the product and Shotgun proteomics. The authors identified 35 bioactive peptides with ACE-inhibitory activity that could be useful for the generation of new drugs and nutraceuticals.

Overall, the ACE-inhibitory activity of the bioactive peptides identified in kefir could be affected by the conditions used during the process of fermentation (i.e., pH, temperature and time) as well as storage conditions of the product. Thus, further work is necessary in order to optimize processing and storage conditions of the product to achieve the highest ACE inhibitory activity.

## 6. In vivo and Ex vivo Studies

In vivo measurements normally refer to record systolic blood pressure in rat models [105,106]. These procedures are based on feeding the animals a standard diet for 12 h and the oral administration of solutions of bioactive peptides at different concentrations [105,106]. The systolic blood pressure of the animals is measured through a non-invasive method, namely tail cuff, that allows researchers to compare the effects of bioactive peptides and captopril or any other antihypertensive drug [105]. ex vivo studies are performed in isolated arteries monitoring the generation of angiotensin II from angiotensin I by ACE in the arterial walls as angiotensin I has no vasoactive impact on its own [107].

Using these in vivo and ex vivo procedures, several bioactive peptides have been identified from dairy products (Table 4). Koyama et al. [108] reported that the oral administration of a peptide derived from milk (KFWGK) had a strong and long-lasting antihypertensive activity in spontaneous hypertensive rats (SHR) with a minimum effective dose of 5 μg/kg. An in vivo study was also conducted to identify cardioprotective peptides from kefir [104]. Three in vivo procedures were conducted in this study including a two-kidney one-clip (2K1C) procedure, plethysmography, and evaluation of ACE activity in blood samples. The authors reported that the treatment with kefir via gavage reduced 37 mmHg of systolic arterial pressure and inhibited 19% of ACE activity.

It is very important to know the mechanism of antihypertensive peptides in the body. Gleeson, Brayden, and Ryan [107] measured the intestinal permeability of two cardioprotective peptides with sequences of Isoleucine–Proline–Proline and Leucine–Lysine–Proline using ex vivo and in vivo models. The authors illustrated that the aforementioned peptides can permeate either through PepT1-mediated uptake or the paracellular route in the small intestine.

In a study conducted by Xue, Wang, Hu, Wu, Wang, Wang, and Yang [115], the authors generated and identified a highly stable (multiple pH and temperature) cardioprotective peptide, namely YQK, from bovine milk casein hydrolyzed by pepsin and trypsin. in vivo experiments revealed the antihypertensive effects of this peptide when administered orally to SHR, significantly reducing the systolic blood pressure of the treated animals. In another study, 20 male SHR were used to assay the cardioprotective effect of three peptides (LIWKL, RPYL, and LNNSRAP) derived from a bovine lactoferrin hydrolysate [116]. The results of this research indicated that the three peptides studied were efficient when decreasing blood pressure in vivo, with the peptide LNNSRAP being the one with the highest antihypertensive activity. The authors also performed an ex vivo analysis using six male New Zealand White rabbits and the results revealed that the peptides RPYL and LIWKL significantly inhibited ACE in comparison with the control sample. In some cases, conflicting results from in vitro and in vivo experiments achieved with bioactive peptides can be observed. For instance, as Geerlings et al. [117] reported, the bioactive peptides with the sequences of TGPIPN, SLPQ, and SQPK from goat milk had higher in vitro IC_50_ compared to the captopril, although the in vivo studies showed similar activities. In such cases and more severe conflict cases, it is necessary to develop more comprehensive and integrated studies accounting for all the available information including in vitro, in vivo, ex vivo, and in silico studies to be able to make decisions based on valid and reproducible data.

## 7. Structure-Function Relationships of Bioactive Peptides

The chemical structure of bioactive peptides for enhanced cardioprotective properties has not been fully elucidated to date. However, several studies have identified few structural features of peptides affecting their ACE inhibitory activity, one of the main mechanisms of the cardioprotective effects of peptides [118,119].

The amino acid sequence and the presence of certain metals can significantly influence the ACE inhibitory activity of bioactive peptides. The presence of hydrophobic amino acids at each of the three C-terminal positions and basic amino acids (lysine, arginine, and histidine) at the C-terminus of the bioactive peptides can significatively increase their ACE inhibitory activity [118,119]. The presence of proline at the C-terminus can lock the -COO group of the peptide into a conformation suitable for interacting with the ACE active site [81,120]. Studies have revealed that the presence of proline at the antepenultimate position can enhance their binding to the ACE active site [81,121]. These structural features were confirmed in the peptides DKIHP and DKIHPF derived from Manchego cheese [122].

**Figure 3:**
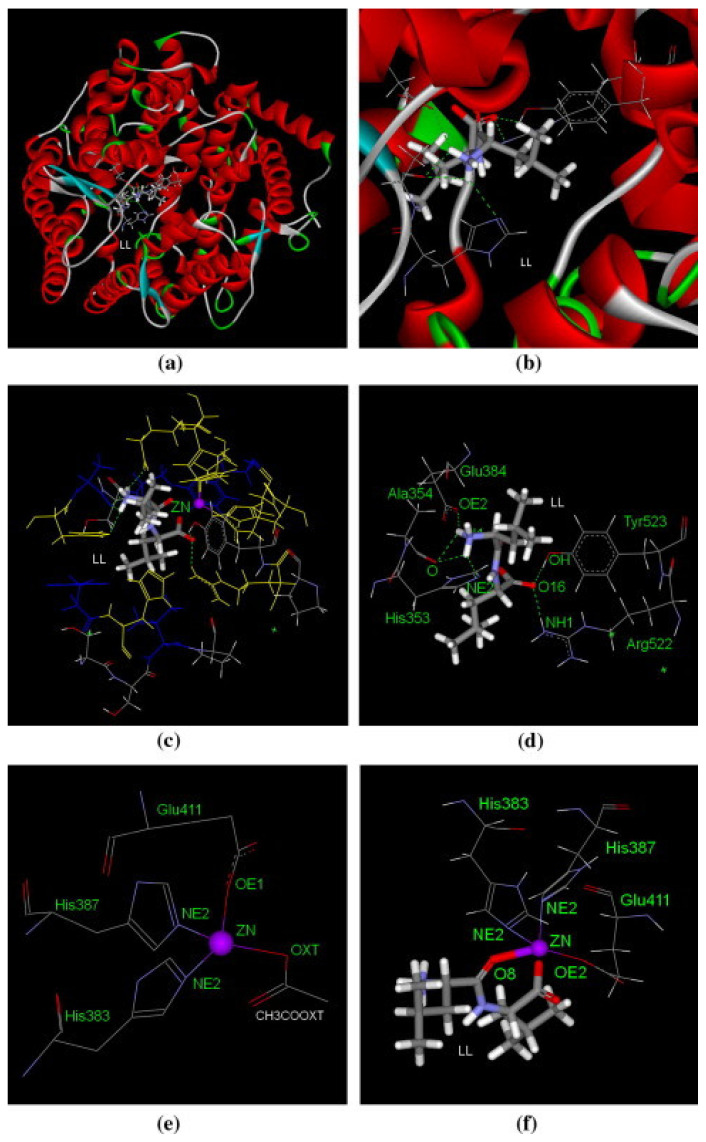
General overview of peptides (LL) interaction with ACE. (**a**) ACE structure and its best docking poses (grey) at the active site, (**b**) regional view of the best docking poses at the active site, (**c**) ACE-LL interaction view at the catalytic site (see the zinc ion in the center of the interactions), (**d**) the best docking poses of LL (grey) at the active site, (**e**) tetrahedrally coordinated zinc ion with the ACE residues before docking, and (**f**) interactions between LL and zinc ion at the ACE active site after docking. ACE hydrophobic and hydrophilic residues are represented in blue and yellow, respectively. Other residues on active site in grey, and zinc atoms in purple. Green dashed lines and grey bold lines represent the hydrogen bonds and zinc coordination bonds, respectively. Reproduced from Pan, Guo, Zhao, and Cao [123] with permission from Elsevier 2011.

Moreover, the presence of Zn^2+^ ion at the ACE active site is also a key factor for the cardioprotective effects of the peptides [123]. To explain this, it is worth noting that the ACE belongs to the category of zinc proteases which means it needs this ion as a cofactor to react with the substrate. The ACE inhibitory peptides can form a distorted geometry surrounding the Zn^2+^ that assists to maintain the peptide–ACE complex leading to a stronger enzyme inhibition [124]. To clarify the structure–function relationship of cardioprotective peptides and their interaction with ACE enzyme several molecular docking techniques can be applied to study these interactions (see Figure 3). During the last few decades, the improvement of molecular modelling innovation has displayed huge progress, particularly since the 2013 Nobel Prize in Chemistry was granted [125]. Among all molecular modelling procedures, molecular docking is the most widely applied method [126].

The process for the generation of novel bioactive peptides with cardioprotective effects includes several steps of isolation and purification before the identification of the compounds, which are laborious and expensive and could also lead to missing some of these compounds during the various activity-based purification steps. Quantitative structure–activity relationship (QSAR) is a novel strategy used to discover novel bioactive peptides with potential cardioprotective effects [127,128]. Furthermore, by gathering information about the structure–function relationship of dairy bioactive peptides, researchers will be capable of designing more potent cardioprotective agents. QSAR is a modelling tool based on chemometrics to elucidate and establish the relationship between chemical structures and their biological activities by using statistical multiple regression analysis, such as partial least squares. Therefore, the biological activity data can be modelled as a function of the structure of the bioactive peptides [129,130]. Several studies have investigated QSAR for the ACE-inhibitory activity [33,34,131,132,133,134,135,136,137,138,139] and renin-inhibitory activity [140] of dairy bioactive peptides. To the best of our knowledge, only a few QSAR studies have been performed on renin-inhibitory bioactive peptides. This could be due to the novelty of this area, as few peptides with renin inhibitory activity have been discovered to date. The results of the QSAR analysis indicated that bioactive peptides with potent renin inhibitory activity contain mainly bulky amino acids (tryptophan, tyrosine, or phenylalanine) at the C-terminus and hydrophobic and small amino acids (valine, leucine, isoleucine, and alanine) at the N-terminus. The presence of tryptophan at the C-terminus of bioactive peptides is predicted to be a key factor affecting their renin inhibitory activity. To confirm this claim, four small bioactive peptides (LW, IW, AW, and VW) have been further studied due to their predicted potent renin inhibitory activities. These biological activities could not be confirmed experimentally for AW and VW; however, the peptides LW and IW were confirmed as powerful inhibitors of renin [128,140].

## 8. Effect of GI Digestion on Bioactive Peptides

In the gastrointestinal tract (GIT), dairy bioactive peptides may be further hydrolyzed by endogenous and microbial proteases. The breakdown of bioactive peptides into smaller peptides and free amino acids improves their bioavailability of these compounds [141]; however, this hydrolysis may reduce the cardioprotective effects of the bioactive peptides [142].

**Figure 4:**
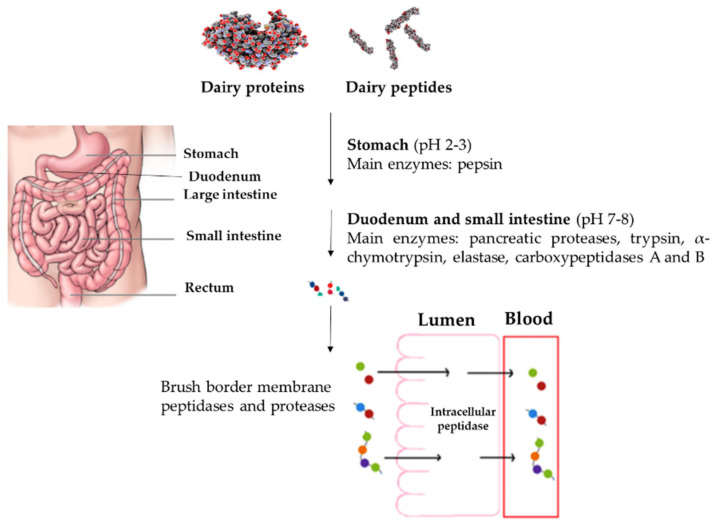
The effects of gastrointestinal tract on dairy proteins and peptides.

The full range of modifications of dairy bioactive peptides passing through GIT is not fully understood yet. It should be considered that there are some changes that may happen to proteins and bioactive peptides structures and biological activities due to the effects of GI juices as well as enzymes as seen in Figure 4. Therefore, GI digestion of dairy products may dramatically affect their biological activities, either by the generation of new bioactive peptides from their original proteins or by further hydrolysis of bioactive peptides, leading to the release of peptide fractions with different biological effects than those pre-digestion [80,91,143].

Different factors affect the resistance of bioactive peptides against GI digestion including their hydrophobicity, acid/base nature, and amino acid composition and peptide sequence, especially the type of C- or N-terminus amino acid residues [144]. Even though hydrophobicity is a key parameter in the ACE-inhibitory activity of bioactive peptides, this factor is indirectly related to the bioactive peptides’ resistance to GI digestion [145]. Similar results associating an increased stability of hydrophilic peptides during GI digestion were reported by Xie et al. [146]. It is also proven that the bioactivity of negatively charged peptides derived from milk is better preserved during passing through GIT followed by positively charged and neutral peptides [145,147]. The development of encapsulation methods for the protection of orally administrated bioactive peptides during GI digestion and the preservation of their biological properties has shown promising results. There are three types of encapsulation methods for bioactive peptides, including protein-based, polysaccharide-based, and lipid-based [148]. Microemulsions and emulsified microemulsions, emulsions, solid lipid particles, liposomes, and polymer microgels have been well established strategies for the protection of bioactive peptides so far [149]. In the case of milk- and dairy-derived peptides, Zhang et al. [150], encapsulated in a liposome the milk-derived ACE inhibitory peptide RLSFNP, for its protection during GI digestion and to enhance its intestinal bioavailability.

## 9. Allergenicity and Toxicity of Bioactive Peptides

Immunoglobulin E (IgE)-mediated reactions are one of the most prominent forms of food allergy; thus, a disturbance of the IgE binding to its epitope could decrease the allergenicity of different compounds [151]. Based on this, treatments such as proteolytic hydrolysis, microbial fermentation, heating, irradiation, and high hydrostatic pressure are currently applied to alleviate the immunoreactivity of certain allergens [152,153,154,155,156].

Proteolytic hydrolysis is currently used in the food industry to prepare hypoallergenic milk formulas [155,157]. Although milk formulas are produced following strict standards, the presence of peptides can stimulate allergic reactions [158,159]. Some allergenic peptides, such as NSAEPEQSLAC, GAQEQNQEQPIRCEKDERF, and VRTPEVDDEAL, have been discovered in these hypoallergenic formulas [147,160]. In a study performed by [161], three peptides derived from milk casein were analyzed for allergenicity using a mast cell allergy model from mouse bone marrow. The results of the incubation of mast cells with peptides indicated the release of histamine and tryptase, markers for mast cells degranulation and amongst all the peptides tested, casomorphin-5 exhibited the maximum allergenic response [161].

The use of certain synthetic drugs to prevent CVD may have adverse secondary effects, thus, dairy products and bioactive peptides have been researched as nutraceuticals against these diseases. Toxicological experiments performed to date revealed no toxicity of dairy bioactive peptides [162,163,164,165]. Maeno, Nakamura, Mennear and Bernard [163] reported that there were no physiological or toxicological changes in-life or postmortem in rats when administering fermented milk containing bioactive peptides. The authors appreciated a single-dose lowest-observable-effect level (LOEL) greater than 4000 mg/kg and a repeated-dose result did not support the identification of a target organ toxicity, with no evidence to support the establishment of a LOEL or maximally tolerated dose (MTD), both being greater than 2 g/kg/day for up to 28 consecutive days [163]. An in vitro study has demonstrated that bioactive peptides derived from dairy-affected malignant cells with no harm to normal cells lines [166]. A study performed by Gleeson et al. [167] revealed that IPP, which is an ACE inhibitory peptide isolated from β-casein, has no cytotoxicity in intestinal (Caco-2) and liver (HepG2) cell lines.

Mutagenicity analysis using bacterial cells are used as a sensitive indirect assay for DNA damage detection [168]. The mutagenicity of a milk-derived peptide (VPP) was tested using *Salmonella* Typhimurium strains TA98, TA100, TA1535, and TA1537 and *Escherichia coli* strain WP2uvrA [169]. The authors reported no DNA damage induced by this antihypertensive peptide. Dairy bioactive peptides not only have shown no genotoxicity, but also in some cases have shown anti-mutagenicity activity [170,171]. Moreover, not toxic signs have been reported for dairy cardioprotective peptides (VPP, IPP, VLSELPEP, LEQVLPRD) using animal models (rats and dogs) in several reports [163,172,173].

## 10. Legislation and Regulatory Requirements for Bioactive Peptides

Functional foods have strong influence on human health beyond their typical dietary characteristics; hence, they ought to be consumed as a component of a healthy diet. In the European Union (EU), one of the main regulations to consider when marketing novel products is the 258/97/CE [174]. This regulation establishes the requirements for the placement in the market of “novel foods”, defined as those that were not used for human consumption to a significant degree within the EU before 15 May 1997 [174]. In the EU, products can be tagged with two classes of claims including health and nutrition. The category of nutritional claims has been established concerning superior nutritional properties, e.g., low fat, high-fiber products, and enriched with health promoting vitamins and amino acids. The relationship between a food/ingredient and human well-being is expressed as a “health claim”. Considering the health claims related to bioactive peptides, there are three clearly distinguished claims including those of basic functions, reduction of the risk of diseases and finally those related to enhancements of children’s health [175]. The abovementioned health claims for improved well-being are well recognized in the case of milk peptides as promoters of the immune system functions [176], egg peptides for lowering the risk of diseases caused by cholesterol [177,178], and fish peptides for developing children’s health [175,179].

To establish claims and get approved on the application of bioactive peptides in food products, the organizations and companies interested in this commercialization need to submit their request to the European Food Safety Authority (EFSA) through an EU member country. Afterwards, the Dietetic Products, Nutrition and Allergies (NDA) panel in the EFSA analyzes the association of bioactive peptides with the claimed health promoting potential based on the regulation of EC (1924/2006) [180], ensuring an effective functioning of the internal EU market, while protecting consumers. Then, following a close investigation of the in vitro and in vivo reliability, potency, safety, dose–response levels, and stability of the products, a decision on these claims is stablished. It is a general agreement among all related researchers and developers that human clinical trials are necessary to ensure the health claims of products containing bioactive peptides. In addition, the EFSA requests information on the applied peptides in food products that includes the peptides’ quantity, peptides’ sequence and length (number of amino acids in peptides sequence), amino acid composition of peptides, molecular weight (MW) distribution, generation procedure, physicochemical characteristics, and conditions to utilize the products [181]. As an example of these legislative procedures in the EU, a product containing cardioprotective peptides with the potential of lowering the blood pressure generated from bonito with the peptide sequence of LKPNM was refused by the EFSA due to insufficient scientific evidence of its efficacy in humans [182].

There are several other parameters that are important for EFSA to standardize a peptide-containing product, including the amount of food vital to achieve the illustrated health benefit, a confident assertion addressing who should not use the product, and a caution notification about the health risks related to excessive consumption [180]. As an example, a market-accessible peptide (Valtyron^®^) derived from sardine with potent ACE inhibitory activity was registered by EFSA. This product must be marked with a label for its specific utilization in some dairy (such as fermented milk, yogurt, powdered milk, and yogurt drink) and non-dairy products (such as beverages, stews, soups, and breakfast cereals) with a maximum consumption limit of 0.6 g per serving.

In the United States of America (USA), one of the largest markets for food products with health benefits, the Food and Drug Administration (FDA) provides a full definition of dietary supplements as “a product (other than tobacco) intended to supplement the diet that bears or contains one or more of the following dietary ingredients: (A) a vitamin; (B) a mineral; (C) an herb or other botanical; (D) an amino acid; (E) a dietary substance for use by man to supplement the diet by increasing the total dietary intake; or (F) a concentrate, metabolite, constituent, extract, or combination of any ingredient described in clause (A), (B), (C), (D), or (E)” (FDA 1994). The terms “functional foods” or “nutraceuticals” are foods regulated under the authority of the Federal Food, Drug, and Cosmetic Act, even though they are not specifically defined by law [183].

**Figure 5:**
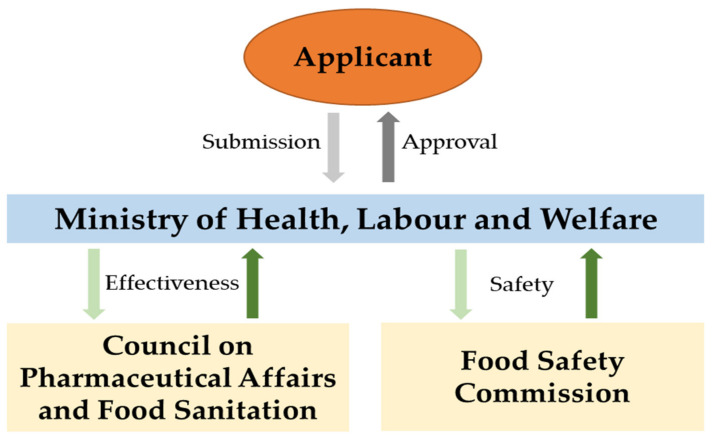
Summary of the procedures for the approval of FOSHU. Content of the image modified from the Japanese Ministry of Health, Labor and Welfare [185].

In the case of Japan, this country has the world’s first policy to allow the commercialization of functional foods, the FOSHU (Foods for Specific Health Use). Established in 1991 by the Ministry of Health, Labor and Welfare, who approved the term, FOSHU is officially used to designate foods that have enough scientific evidence to support health claims [184]. The overall process for approval of FOSHU is summarized in Figure 5.

In Japan, there are some commercially available functional products containing bioactive peptides approved by FOSHU. These products include sour milk, milk casein hydrolysate, Katsuobushi oligopeptide, Mycoleptodonoide saitchisonii extract, bioactive peptide derived from sardine, seaweed, and sesame, all with cardioprotective effects via ACE inhibition. To the best of our knowledge, there are a few products containing cardioprotective peptides derived from milk that are listed in the Table 5. Further research is still required to clarify the exact mechanism and intake efficiency of these antihypertensive peptides [186].

## 11. Future Perspective and Conclusions

Based on the current scientific evidence provided in different studies, milk from multiple animal species, including cow, goat, camel and others, have the potential to produce multiple cardioprotective peptides via direct processing of milk (fermentation and enzymatic hydrolysis) or by more complex food processes, such as the production of yogurt, cheese, and Kefir. The animal species of origin as well as the processing and storage of products will contribute to the variability of peptides present in these products and, thus, their biological and cardioprotective properties.

Further studies are also needed to evaluate the presence of bioactive peptides in multiple dairy food products as well as to evaluate the effect of the gastrointestinal digestion on the identified peptide sequences. Gastrointestinal digestion of different peptides results in smaller peptides sequences that may have an increased bioavailability [141], but the biological properties of these compounds may be altered or even reduced [142]. The evaluation of the cardioprotective effects of the identified peptides using in vitro, ex vivo and in vivo models, including clinical trials, will be crucial to understand the structure–function relationship of these peptides with their target molecules, establishing clear mechanisms of action that will aid companies and producers to establish clear health claims to market the products following the specifications of the various legislative frameworks available worldwide.

Overall, although further studies on the toxicity and allergenicity of these compounds will still be necessary to establish the safety of each specific product and their recommended/tolerable intakes aiming their safe commercialization, several milk-derived peptides evaluated to date have been shown to be safe, opening a promising scenario for the development and further evaluation of milk-derived products containing bioactive peptides.

## Figures and Tables

**Table 1:** Cardioprotective peptides derived from milk using enzymatic proteolytic procedures.

Original Proteins	Proteolytic Procedures	Peptide Sequences	Cardioprotective Mechanisms	References
Bovine casein	Peptidase from *Maclura pomifera* latex	YQEPVLGPVRGPFPIIV and RFFVAPFPE	ACE inhibitory	[35]
Goat milk	Simulated gastro-intestinal digestion	AEK, AI, IPP, AY, andVP	ACE inhibitory	[36]
Buffalo skimmed milk	Papain, pepsin, or trypsin	FPGPIPK, IPPK, IVPN, and QPPQ	ACE inhibitory	[37]
Camel milk casein	Pepsin, trypsin, and chymotrypsin	Not identified	ACE inhibitory	[38]
Camel milk	Simulated gastro-intestinal digestion	IPP	ACE inhibitory	[39]
Defatted milk powder	Continuous enzyme membrane reactor (EMR)	Not identified	ACE inhibitory	[24]
Camel milk whey hydrolysates	Pepsin	PVAAAPVM and LRPFL	Renin inhibitory and ACE inhibitory	[40]
Caprine milk	Neutral protease and pepsin	Not identified	ACE inhibitory	[41]
Yak milk casein	Screened using quantitative structure-activity relationship (QSAR) models	KYIPIQ	Production of nitric oxide by ACE inhibition	[42]
Milk protein concentrate	Alcalase, protamex, flavourzyme, proteAXH, and protease A2SD	QEPVLGPVRGPFP and YPFPGPIPN	ACE inhibitory	[43]

**Table 3:** Bioactive peptides with potential cardioprotective effects derived from cheese.

Cheese Type	Protein	Culture	Peptide Sequence	References
Cheddar	κ-CN	*Lactococcus lactis* subsp. *lactis* and *L. lactis* subsp. *cremoris* with adjunct culture of *Lactobacillus acidophilus* LAFTI^®^ L10	ARHPHPH	[89]
	α_s1_-CN		RPKHPIKHQ	
	α_s1_-CN		RPKHPIK	
	α_s1_-CN		RPKHPI	
	α_s1_-CN		FVAPFPEVF	
	β-CN		YQEPVLGPVRGPFPIIV	
White brined-cheese	Casein	*L. lactis* subsp. *lactis* and *L. lactis* subsp. *cremoris* with adjunct culture of *Lactobacillus helveticus*	Not identified	[90]
Valdeón	α_s1_-CN β-CN	*Penicillium roqueforti*	DAYPSGAW DKIHPF	[91]
Grana Padano	β-CN	*L. helveticus* is added to raw milk prior to renneting	HLPLP	[92]
Parmigiano Reggiano	β-CN β-CN β-CN	Natural whey starter prior to renneting	VPP IPP LHLPLP HLPLP	[93]
Prato	α_s1_-CN	*L. lactis* subsp. *lactis* and *L. lactis* subsp. *cremoris*	FVAPFPEVF	[94]

**Table 4:** In vivo and ex vivo studies assessing cardioprotective properties of peptides from dairy.

Peptides Sequences	Original Proteins	Methods-Models	Cardioprotective Results	References
LRPVAA	Whey protein (Lactoferrin)	In vivo—SHR *	1 nmol/kg body weight of peptides decreased systolic pressure of SHR	[109]
FKCRRWQWRMKKLGA	Whey protein (lactoferricin B_17-31_)	Ex vivo—RCAS **	20 µM of peptides inhibited ACE-dependent angiotensin I-induced contraction	[110]
NI	Whey protein (Lactoferrin)	Ex vivo—RCAS ***	4.49 mg/mL of peptides inhibited ACE-dependent angiotensin I-induced contraction	[111]
RRWQWR and WQ	Whey protein (lactoferricin B_20-25_)	In vivo—SHR	10 mg/kg body weight of peptides decreased systolic pressure of SHR	[112]
FKCRRWQWRMKKLGAP RRWQWR CRRWQWR KCRRWQWR FKCRRWQWR FKCRRWQW FKCRRW	Whey protein (lactoferricin B_17-32_) (lactoferricin B_20-25_) (lactoferricin B_19-25_) (lactoferricin B_18-25_) (lactoferricin B_17-25_) (lactoferricin B_17-24_) (lactoferricin B_17-22_)	Ex vivo—RCAS	20 µM of peptides inhibited ACE-dependent angiotensin I-induced contraction	[112]
HLPLP, HLPL, LPLP and HLP	Milk (β-casein)	In vivo—SHR	7 mg/kg body weight of peptides decreased systolic pressure of SHR	[113]
IPP and LKP	Milk	In vivo—Isoflurane-anaesthetised rats intra-jejunal Ex vivo—isolated jejunal tissue from Isoflurane-anaesthetised rats	24 µM of peptides showed transepithelial permeability 3 µM of peptides showed transepithelial permeability	[107]
IW IW, EW and WL	Milk	In vivo—SHR Ex vivo—isolated arota of male Wistar rats	19 mg/kg body weight of peptides decreased systolic pressure of SHR 10 µM of peptides decreased the angiotensin I/angiotensin II ratio	[114]
YQK	Milk (casein)	In vivo—SHR	1–9 19 mg/kg body weight of peptides decreased systolic pressure of SHR	[115]

* Spontaneously hypertensive rats. ** Rabbit carotid arterial segments. *** Not indicated.

**Table 5:** Commercial products containing cardioprotective peptides derived from milk.

Item Names	Type	Peptides Sequences	Manufacturers	References
Calpis	Sour milk	IPP and VPP	Calpis Co., Ltd., Tokyo, Japan	Patent EP0323283
Evolus	Fermented milk	IPP and VPP	Valio, Ltd., Helsinki, Finland	Patent US6972282
Amealbp	Tablets	IPP and VPP	Calpis Co., Ltd., Tokyo, Japan	
Ameal Peptide	Ingredient	IPP and VPP	Calpis Co., Ltd., Tokyo, Japan	
Casein DP	Soft drink	FFVAPFPEVFGK	Kanebo Co., Ltd., Tokyo, Japan	Patent JP62270533
C12 Peptide	Ingredient	FFVAPFPEVFGK	DMV International, Veghel, Netherlands	
Lowpept	Ingredient	RYLGY and AYFYPEL	Innaves S.A., Pontevedra, Spain	Patent WO012355
BioZate 1	Ingredient	β-lactoglobulin fragments	Davisco Foods, Minnesota, USA	Patent US6998259

## Data Availability

The data presented in this study are available in the article.
